# Oral health and later coronary heart disease: Cohort study of one million people

**DOI:** 10.1177/2047487318759112

**Published:** 2018-02-20

**Authors:** G David Batty, Keum Ji Jung, Yejin Mok, Sun Ju Lee, Joung Hwan Back, Sunmi Lee, Sun Ha Jee

**Affiliations:** 1 Department of Epidemiology and Public Health, University College London, UK; 2 Department of Epidemiology and Health Promotion, Yonsei University, South Korea; 3 Institute for Health Promotion, Yonsei University, South Korea; 4 Department of Epidemiology, Johns Hopkins Bloomberg School of Public Health, USA; 5 Health Insurance Policy Research Institute, Wonju, South Korea

**Keywords:** Oral health, coronary heart disease, epidemiology

## Abstract

**Aims:**

Systematic reviews report an association between poorer oral health and an increased risk of coronary heart disease. This contentious relationship may not be causal but existing studies have been insufficiently well powered comprehensively to examine the role of confounding, particularly by cigarette smoking. Accordingly, we sought to examine the role of smoking in generating the relationship between oral health and coronary heart disease in life-long non-smokers.

**Methods and results:**

In the Korean Cancer Prevention Study, 975,685 individuals (349,579 women) aged 30–95 years had an oral examination when tooth loss, a widely used indicator of oral health, was ascertained. Linkage to national mortality and hospital registers over 21 years of follow-up gave rise to 64,784 coronary heart disease events (19,502 in women). In the whole cohort, after statistical adjustment for age, there was a moderate, positive association between tooth loss and coronary heart disease in both men (hazard ratio for seven or more missing teeth vs. none; 95% confidence interval 1.08; 1.02, 1.14; *P*_trend_ across tooth loss groups <0.0001) and women (1.09; 1.01, 1.18; *P*_trend_ 0.0016). Restricting analyses to a subgroup of 464,145 never smokers (25,765 coronary heart disease events), however, resulted in an elimination of this association in men (1.01; 0.85, 1.19); *P*_trend_ 0.7506) but not women (1.08; 0.99, 1.18; *P*_trend_ 0.0086).

**Conclusion:**

In men in the present study, the relationship between poor oral health and coronary heart disease risk appeared to be explained by confounding by cigarette smoking so raising questions about a causal link.

## Introduction

Around a century ago, the theory of focal infection posited that dental caries were aetiologically linked to an array of pathological conditions in distal organs, particularly the heart.^1^ Still a source of contemporary debate, the number of observational studies – cross-sectional, case–control and cohort – directly examining the link between periodontal disease, broadly defined, and coronary heart disease has risen exponentially in the last two decades. Systematic reviews of this evidence base,^2–6^ most recently including an American Heart Association scientific statement,^7^ suggest that poor oral health assessed using self-report or clinical examination is related to an elevated risk of coronary heart disease. Mechanistic support for this association has been found in studies demonstrating that local oral bacterial infection is associated with increased systemic inflammatory activity,^8^ which has itself been implicated in the aetiology of coronary heart disease.^9^ If causally linked, then the high occurrence of both periodontal disease and coronary heart disease – vascular disease is a leading cause of morbidity burden^10^ and periodontal disease prevalence is high in selected groups (e.g. people with diabetes)^11^ – raises the possibility that treating periodontal disease more systematically and aggressively could lead to a marked reduction in coronary heart disease rates.

With the extant evidence for a relationship between oral health and coronary heart disease events being based exclusively on observational data, a key issue in data interpretation is the perennial problem of confounding.^7^ That is, characteristics related to both periodontal disease and coronary heart disease – alcohol intake, diabetes mellitus, low socioeconomic status and, particularly, smoking – provide an alternative explanation for the association. In the current and probable future lack of any aetiologically orientated randomised controlled trial with the capacity to examine if the reversal of oral disease by conventional treatment causes a concomitant reduction in the risk of incident coronary heart disease, investigators typically control for candidate confounding variables in their observational studies in an effort to identify ‘independent’ effects.^7^ In general, the relation between tooth loss and coronary heart disease appears to be robust to such statistical adjustments.

While statistical control for covariates is the most commonly utilised technique for exploring ‘independent’ effects for oral health on coronary heart disease, this approach inevitably raises concerns regarding residual confounding – that is, the failure to fully characterise an individual for a given exposure across the life course. An alternative and perhaps more powerful approach is to explore the tooth loss–coronary heart disease link in people who do not drink, or smoke, or are free of diabetes in which any confounding structure is essentially broken. Intriguingly, in a small cohort of older never-smoking people – to our knowledge, the only prospective study to explore the link in a non-patient population – there was no association between tooth loss and incident coronary heart disease.^12^ With the apparent link between oral disease and coronary heart disease remaining a source of interest, we utilised a well characterised, general population-based sample of around one million men and women, over 450,000 of whom had never smoked.

## Methods

The Korean Cancer Prevention Study is a prospective cohort study established to identify environmental risk factors for major causes of mortality, particularly malignancy, in an east Asian population. Described in detail elsewhere,^13–15^ the cohort comprises government employees and their dependents who were registered with the Korean Medical Insurance Corporation. In generating the present study sample, we applied the following inclusion criteria: (a) member of Korea Medical Insurance Corporation between 1992 and 1995; (b) participation in at least one routine medical examination during this period which included completion of a medical examination and lifestyle questionnaire; and (c) 30 years of age or older at first measurement. A total of 1,329,525 people (482,618 women) met these inclusion criteria and this group comprises the present study. The institutional review boards of Yonsei University and the Johns Hopkins University Bloomberg School of Public Health approved the study. That analyses were based on anonymised data meant that individual study member consent was not required.

### Examination of the oral cavity

An examination of the oral cavity was carried out by a dental physician during which a count was made of the number of natural teeth present in the mouth.^16^ Artificial teeth were not included, but any tooth or part of a tooth that was visible in the mouth and connected to the gum or jawbone was counted as a tooth. The number of missing teeth was our measure of oral disease in the present study; the higher the number, the greater the assumed severity of oral disease. Tooth loss is a commonly used indicator of oral disease in population-based studies.^7^

### Potential confounding variables

Blood samples were obtained after an overnight fast and assayed for glucose and cholesterol using standard protocols. Based on existing definitions,^17^ diabetes mellitus was denoted by a blood glucose level of 126 mg/dl or greater and/or self-report of either physician diagnosis or medication usage. Each hospital used for blood analyses followed the quality control procedures of the Korean Association of Laboratory Quality Control. The weight and height of each study member was measured directly in light clothing with shoes removed, and body mass index was calculated in the usual manner (weight in kilograms divided by height in meters squared).

With the study member present, completed questionnaires were scrutinised and, when necessary, clarification sought. Smoking (current smokers, former and never) and exercise (yes, no) were self-reported as was current total daily alcohol consumption, which was expressed as the number of glasses per week of ‘soju’. Comparable to vodka, soju is the most popular alcoholic beverage in Korea, with one glass containing about 12 g of ethanol. Alcohol consumption was categorised as follows: non-drinker (0 g/per day); light drinker (1–24.9); moderate drinker (25–49.9); heavy drinker (50–99); and very heavy drinker (>99). Available in a subgroup of study members (*N* = 1,034,768), our measure of socioeconomic status was the monetary contribution per year, in South Korean ‘Won’ (1112 Won = US $1.00), made by the employee to their medical insurance scheme. This is means derived, being based on the employee’s income plus assets, such as ownership of property and an automobile. A higher employee contribution to the medical scheme therefore denotes higher socioeconomic status.

### Ascertainment of coronary heart disease mortality and morbidity

Non-fatal and fatal outcomes were ascertained from health insurance claims and death certificates, respectively. Coronary heart disease events extracted from insurance claims show a reasonable level of agreement with hospital records.^18^ Computerised searches for death certificates were performed using a national identification number assigned at birth by the National Statistical Office. Trained recorders extracted the cause of death according to the International Classification of Diseases, 10th revision (ICD-10, I20-I25).^19^ Event surveillance began on 1 January 1993 with study members being censored on the date of the coronary heart disease event or end of follow-up (31 December 2013) whichever came first.

### Statistical analyses

Participants who died before 1st January 1993 were excluded from analyses (*n* = 1714). In an attempt to avoid reverse causality due to current disease – existing illness could influence tooth loss rather than the reverse – we further excluded 50,675 participants with a known history of cancer, cardiovascular disease, liver disease and/or a respiratory disease as determined at the initial medical examination. After additional exclusions of study members with missing data, the current analysis was based on an analytical sample of 975,685 individuals (349,579 women).

Having first determined that the proportional hazards assumption had not been violated for tooth loss in relation to coronary heart disease, we used Cox models to compute hazard ratios with accompanying 95% confidence intervals for men and women separately.^20^ Hazard ratios for the tooth loss–coronary heart disease association were adjusted for age, and then separately for other covariates (socioeconomic status, height, alcohol intake, smoking status, exercise, systolic blood pressure, fasting blood cholesterol, diabetes, body mass index and family history of cardiovascular disease). All analyses were conducted using SAS version 9.2 (SAS Institute Inc., Cary, NC, USA).

## Results

Having any tooth loss (one or more tooth) was twice as common in men (30%) relative to women (15%). Gender differentials in average alcohol consumption (17.2 vs. 0.2 g/day) and cigarette smoking prevalence (58.2% vs. 3.4%) were also stark, with much higher levels seen in men. In Tables 1 (men) and 2 (women) we show the distribution of study covariates according to categories of tooth loss. In general, study members with some degree of toothlessness had a less favourable risk factor profile. Although these differences often achieved statistical significance at conventional levels, this was due to the high sample size with the absolute differences often being small. Thus, in men, relative to the dentate, those in the greatest tooth loss category were older, somewhat more likely to be socioeconomically disadvantaged, shorter in physical stature, more likely to be a smoker, have higher systolic blood pressure, have slightly higher fasting blood glucose, have more than twice the prevalence of diabetes, and were more likely to have an increased genetic predisposition to heart disease as indicated by a family history. With the exception of age and smoking, however, several of these differences across the tooth loss groups were not incremental. There was some suggestion of a higher intake of alcohol in the men with greater tooth loss. Conversely, men with greater tooth loss were less likely to be sedentary. In women, many of these differences in levels of covariates across tooth loss categories were also apparent, although, again, differences across tooth loss groups were not always marked nor stepwise.

**Table 1.** Baseline characteristics according to tooth loss in 626,106 men in the Korean Cancer Prevention Study.

	Number of missing teeth (number of study members)				*P* value for trend	All study members
	0 (439,102)	1–3 (151,413)	4–6 (24,311)	≥7 (11,280)		
Age (year), mean (SD)	44.0 (10.8)	45.4 (9.1)	50.0 (8.8)	55.3 (9.0)	<0.0001	44.8 (10.5)
Number of missing teeth, mean (SD)	0.0 (0.0)	1.6 (0.7)	4.6 (0.8)	11.8 (5.9)	<0.0001	0.8 (2.0)
SES (insurance contribution, Won × 10^3^), mean (SD)	116.9 (75.8)	106.8 (71.2)	102.9 (74.6)	107.4 (75.9)	<0.0001	113.7 (75.0)
Height (cm), mean (SD)	168.8 (5.4)	168.7 (5.2)	167.8 (5.4)	166.6 (5.7)	<0.0001	168.7 (5.3)
Alcohol intake (g/day), mean (SD)	16.3 (30.1)	19.9 (35.0)	19.1 (37.3)	14.4 (33.4)	<0.0001	17.2 (31.7)
Current smoker, % (*N*)	56.1 (246,267)	62.6 (94,777)	66.0 (16,037)	65.4 (7373)	<0.0001	58.2 (364,454)
No exercise, % (*N*)	71.5 (313,730)	70.7 (107,048)	68.7 (16,710)	66.4 (7487)	<0.0001	71.1 (444,975)
Systolic blood pressure (mmHg), mean (SD)	123.5 (15.4)	125.0 (15.7)	127.5 (17.3)	128.4 (18.6)	<0.0001	124.1 (15.6)
Fasting blood cholesterol (mg/dL), mean (SD)	190.9 (37.2)	193.0 (38.2)	194.0 (38.4)	193.9 (39.4)	<0.0001	191.6 (37.5)
Diabetic, % (*N*)	3.9 (17,290)	5.1 (7727)	7.2 (1740)	9.5 (1066)	<0.0001	4.4 (27,823)
Body mass index (kg/m^2^), mean (SD)	23.2 (2.5)	23.4 (2.5)	23.2 (2.6)	22.8 (2.6)	0.2220	23.3 (2.5)
Family history of cardiovascular disease, % (*N*)	15.7 (58,827)	15.0 (19,488)	14.3 (2920)	13.1 (1200)	<0.0001	15.4 (82,435)

**Table 2.** Baseline characteristics according to tooth loss in 349,579 women in the Korean Cancer Prevention Study.

	Number of missing teeth				*P* value for trend	All study members
	0 (296,754)	1–3 (37,019)	4–6 (8417)	≥7 (7389)		
Age (year), mean (SD)	48.6 (11.5)	45.7 (10.1)	51.4 (10.1)	58.6 (9.4)	<0.0001	48.6 (11.4)
Number of missing teeth, mean (SD)	0.0 (0.0)	1.6 (0.7)	4.7 (0.8)	13.8 (6.9)	<0.0001	0.6 (2.4)
SES (insurance contribution, Won × 10^3^), mean (SD)	115.1 (71.4)	114.5 (70.6)	110.6 (69.8)	111.2 (60.5)	<0.0001	114.8 (71.1)
Height (cm), mean (SD)	155.1 (5.5)	156.1 (5.2)	154.6 (5.6)	152.3 (5.7)	<0.0001	155.1 (5.5)
Alcohol intake (g/day), mean (SD)	0.2 (1.8)	0.3 (2.3)	0.2 (2.2)	0.2 (2.0)	<0.0001	0.2 (1.9)
Current smoker, % (*N*)	3.5 (10,240)	1.8 (655)	3.6 (304)	7.9 (586)	<0.0001	3.4 (11,785)
No exercise, % (*N*)	83.1 (246,710)	83.1 (30,744)	81.1 (6827)	80.6 (5958)	<0.0001	83.0 (290,239)
Systolic blood pressure (mmHg), mean (SD)	120.9 (18.7)	119.2 (17.2)	123.2 (19.1)	126.8 (20.7)	<0.0001	120.9 (18.7)
Fasting blood cholesterol (mg/dL), mean (SD)	194.2 (39.1)	192.0 (39.0)	196.9 (39.4)	200.5 (39.1)	<0.0001	194.1 (39.2)
Diabetic, % (*N*)	3.7 (10,903)	2.9 (1068)	4.5 (381)	6.8 (504)	<0.0001	3.7 (12,856)
Body mass index (kg/m^2^), mean (SD)	23.2 (3.1)	23.0 (3.1)	23.7 (3.1)	23.8 (3.2)	<0.0001	23.2 (3.1)
Family history of cardiovascular disease, % (*N*)	18.6 (45,891)	19.5 (5885)	18.0(1185)	15.9(874)	0.0311	18.6 (53,835)

Twenty-one years of follow-up gave rise to 64,784 coronary heart disease events (3364 deaths, 61,420 hospitalisations). In Tables 3 (men) and 4 (women) we show the relation of tooth loss to the future occurrence of coronary heart disease. In men, tooth loss was associated with an elevated risk of coronary heart disease. The magnitude of these relationships was, however, modest such that the greatest increased risk associated with tooth loss was around 10%. There was some evidence that adjustment for covariates had a partial attenuating effect, particularly following control for behavioural factors which included cigarette smoking. We made similar observations to these in women (Table 4). In order to search for any inflections in the tooth loss–coronary heart disease relationship that might be masked by using broader categories of our exposure, we utilised the full range of tooth loss values and repeated our analyses (see Supplementary Figure 1). There was no clear evidence of a threshold effect.

**Table 3.** Hazard ratio (95% confidence intervals) for the relation of baseline tooth loss with later coronary heart disease in 626,106 men in the Korean Cancer Prevention Study.

	*N* events/ *N* at risk	Number of missing teeth				*P* value for trend	Per 2 teeth lost
		0	1–3	4–6	≥7		
Number of people at risk	45,282/626,106	439,102	151,413	24,311	11,280	–	–
Number of coronary heart disease events	45,282/626,106	29,876	11,972	2258	1176	–	–
Age	45,282/626,106	1.0 (ref)	1.07 (1.05, 1.10)	1.08 (1.04, 1.13)	1.08 (1.02, 1.14)	<0.0001	1.02 (1.01, 1.02)
Age and social factors	45,282/626,106	1.0	1.08 (1.05, 1.10)	1.09 (1.04, 1.13)	1.08 (1.02, 1.15)	<0.0001	1.02 (1.01, 1.03)
Age and behavioural factors	45,282/626,106	1.0	1.06 (1.04, 1.08)	1.05 (1.01, 1.10)	1.04 (0.98, 1.10)	<0.0001	1.01 (1.00, 1.02)
Age and physiological factors	38,540/535,317	1.0	1.04 (1.02, 1.07)	1.05 (1.00, 1.10)	1.09 (1.02, 1.17)	<0.0001	1.02 (1.01, 1.03)
Multiple adjustment	38,540/535,317	1.0	1.03 (1.01, 1.05)	1.02 (0.97, 1.07)	1.05 (0.99, 1.12)	0.0136	1.01 (1.00, 1.02)
Multiple adjustment with exclusions^a^	36,527/529,069	1.0	1.03 (1.01, 1.05)	1.02 (0.97, 1.07)	1.03 (0.97, 1.11)	0.0381	1.01 (1.00, 1.02)

Social factors: socioeconomic status, height.

Behavioural factors: Alcohol intake, smoking status, exercise.

Physiological factors: systolic blood pressure, fasting blood cholesterol, diabetes, body mass index, family history of cardiovascular disease.

Multiple adjustment is adjustment for all above covariates.

a Excluding coronary heart disease events in the first 5 years of follow-up.

**Table 4.** Hazard ratio (95% confidence intervals) for the relation of baseline tooth loss with later coronary heart disease in 349,579 women in the Korean Cancer Prevention Study.

	*N* events/ *N* at risk	Number of missing teeth				*P* value for trend	Per 2 teeth lost
		0	1–3	4–6	≥7		
Number of people at risk	19,502/349,579	296,754	37,019	8417	7389	–	–
Number of coronary heart disease events	19,502/349,579	16,486	1,791	575	650	–	–
Age	19,502/349,579	1.0 (ref)	1.02 (0.97, 1.07)	1.12 (1.03, 1.22)	1.09 (1.01, 1.18)	0.0016	1.01 (1.00, 1.02)
Age and social factors	19,502/349,579	1.0	1.02 (0.97, 1.07)	1.12 (1.03, 1.22)	1.09 (1.01, 1.18)	0.0015	1.01 (1.00, 1.02)
Age and behavioural factors	19,502/349,579	1.0	1.03 (0.98, 1.08)	1.13 (1.04, 1.22)	1.09 (1.01, 1.18)	0.0012	1.01 (1.00, 1.02)
Age and physiological factors	15,809/288,915	1.0	1.00 (0.95, 1.06)	1.08 (0.98, 1.18)	1.10 (1.01, 1.21)	0.0201	1.01 (1.00, 1.02)
Multiple adjustment	15,809/288,915	1.0	1.01 (0.96, 1.07)	1.08 (0.99, 1.19)	1.11 (1.01. 1.21)	0.0122	1.01 (1.00, 1.02)
Multiple adjustment with exclusions^a^	14,804/285,927	1.0	1.00 (0.94, 1.06)	1.08 (0.98, 1.19)	1.05 (0.95, 1.16)	0.1686	1.01 (1.00, 1.02)

Social factors: socioeconomic status, height.

Behavioural factors: Alcohol intake, smoking status, exercise.

Physiological factors: Systolic blood pressure, fasting blood cholesterol, diabetes, body mass index, family history of cardiovascular disease.

Multiple adjustment is adjustment for all above covariates.

a Excluding coronary heart disease events in the first 5 years of follow-up.

Next, in Tables 5 (men) and 6 (women) we present the relation of tooth loss to the future occurrence of coronary heart disease according to subgroups of different confounding variables. In men who were non-drinkers, without diabetes, and were advantaged socioeconomically, the observation of a positive tooth loss–coronary heart disease relation was again apparent. In life-long never smoker men, however, the association was essentially lost with all point estimates around unity. In women, the general pattern of an increased risk of coronary heart disease with a greater degree of tooth loss remained, even in never smokers.

**Table 5.** Age-adjusted hazard ratios (95% confidence intervals) for the relation of baseline tooth loss with later coronary heart disease in 626,106 men by strata of health behaviours, diabetes, and socioeconomic status in the Korean Cancer Prevention Study.

Analytical group (no. events/no. at risk)	Number of missing teeth				*P* value for trend	Per two teeth tooth lost
	0	1–3	4–6	≥7		
Whole cohort (45,282/626,106)	1.0 (ref)	1.07 (1.05, 1.10)	1.08 (1.04, 1.13)	1.08 (1.02, 1.14)	<0.0001	1.02 (1.01, 1.02)
Never smoker (8062/132,454)	1.0	0.99 (0.94, 1.05)	0.98 (0.87, 1.10)	1.01 (0.85, 1.19)	0.7506	1.00 (0.98, 1.03)
Former smoker (9,689/129,198)	1.0	1.04 (0.99, 1.09)	1.08 (0.98, 1.19)	1.02 (0.90, 1.16)	0.0698	1.01 (0.99, 1.02)
Current smoker (27,531/364,454)	1.0	1.08 (1.05, 1.11)	1.06 (1.00, 1.11)	1.06 (0.98, 1.14)	<0.0001	1.01 (1.00, 1.02)
Non-drinkers (12,698/144,723)	1.0	1.09 (1.05, 1.14)	1.13 (1.04, 1.22)	1.10 (1.00, 1.22)	<0.0001	1.02 (1.01, 1.03)
Drinkers (32,584/481,383)	1.0	1.07 (1.05, 1.10)	1.07 (1.02, 1.13)	1.07 (0.99, 1.15)	<0.0001	1.02 (1.00, 1.03)
Non-diabetics (41,250/598,283)	1.0	1.06 (1.04, 1.09)	1.06 (1.01, 1.11)	1.06 (1.00, 1.13)	<0.0001	1.01 (1.01, 1.02)
Diabetics (4032/27,823)	1.0	1.06 (0.99, 1.14)	1.09 (0.96, 1.24)	1.04 (0.89, 1.22)	0.0959	1.02 (0.99, 1.04)
High socioeconomic status (12,315/159,451)	1.0	1.05 (1.01, 1.09)	1.06 (0.98, 1.14)	1.04 (0.93, 1.16)	0.0280	1.01 (1.00, 1.03)
Low socioeconomic status (32,967/466,655)	1.0	1.09 (1.06, 1.12)	1.10 (1.04, 1.15)	1.10 (1.03, 1.18)	<0.0001	1.02 (1.01, 1.03)

**Table 6.** Age-adjusted hazard ratios (95% confidence intervals) for the relation of baseline tooth loss with later coronary heart disease in 349,579 women by strata of health behaviours, diabetes, and socioeconomic status in the Korean Cancer Prevention Study.

Analytical group (no. events/no. at risk)	Number of missing teeth				*P* value for trend	Per two teeth tooth lost
	0	1–3	4–6	≥7		
Whole cohort (19,502/349,579)	1.0 (ref)	1.02 (0.97, 1.07)	1.12 (1.03, 1.22)	1.09 (1.01, 1.18)	0.0016	1.01 (1.00, 1.02)
Never smoker (17,703/331,691)	1.0	1.01 (0.96, 1.07)	1.11 (1.02, 1.21)	1.08 (0.99, 1.18)	0.0086	1.01 (1.00, 1.02)
Former smoker (523/6103)	1.0	1.10 (0.75, 1.59)	1.04 (0.60, 1.81)	1.18 (0.80, 1.76)	0.3765	1.02 (0.97, 1.06)
Current smoker (1276/11,785)	1.0	1.22 (0.97, 1.54)	1.25 (0.91, 1.72)	1.07 (0.84, 1.37)	0.1621	1.01 (0.98, 1.04)
Non-drinkers (17,053/300,415)	1.0	1.02 (0.97, 1.08)	1.11 (1.01, 1.21)	1.08 (0.99, 1.18)	0.0080	1.01 (1.00, 1.02)
Drinkers (2449/49,164)	1.0	1.02 (0.88, 1.16)	1.23 (0.99, 1.53)	1.16 (0.95, 1.43)	0.0457	1.02 (0.99, 1.04)
Non-diabetics (17,721/336,723)	1.0	1.03 (0.98, 1.09)	1.12 (1.03, 1.23)	1.09 (1.00, 1.19)	0.0015	1.01 (1.00, 1.02)
Diabetics (1781/12,856)	1.0	0.92 (0.77, 1.09)	1.03 (0.79, 1.34)	1.00 (0.79, 1.27)	0.8317	1.00 (0.97, 1.03)
High socioeconomic status (4582/84,506)	1.0	1.00 (0.90, 1.10)	1.04 (0.88, 1.24)	1.13 (0.95, 1.34)	0.2098	1.01 (0.99, 1.04)
Low socioeconomic status (14,920/265,073)	1.0	1.03 (0.97, 1.09)	1.15 (1.04, 1.26)	1.08 (0.99, 1.18)	0.0043	1.01 (1.00, 1.02)

## Discussion

The aim of the present analyses was to explore alternative explanations for an association between poorer oral health and raised coronary heart disease risk. In the full cohort of men and women we found a modest age-adjusted relationship between tooth loss and coronary heart disease, such that in people in the highest tooth loss group (seven or more) had around a 10% elevated risk of coronary heart disease. After control for potential confounding factors, particularly smoking, there was partial attenuation of this gradient. In subgroup analyses of never smoking men, however, there was no apparent tooth loss–coronary heart disease relationship. The corresponding analyses in women resulted in the tooth loss–coronary heart disease gradient being essentially unaltered. We are unaware of any biologically or socially plausible explanation for smoking not being a confounding variable in women as it appears to be in men, particularly when, in women, the smoking–coronary heart disease gradient appears to be somewhat steeper than for men.^21^

### Existing evidence

While numerous studies have explored the link between tooth loss and coronary heart disease, analyses of never smokers or even current non-smokers is rare, particularly in disease-free men and women, not least because very large scale studies are required. In a cohort of patients with a history of myocardial infarction,^22^ there was a positive relation between tooth loss and disease recurrence in never smokers. It is unclear, however, if the same disease processes that underlie a link between tooth loss and a second myocardial event are the same as those for incident cases. In a study of cardiovascular disease-free individuals in which stroke was the endpoint of interest,^23^ men who were seropositive for *Porphyromonas gingivalis*, one of two serum antibodies tested, had an elevated rate of stroke even when analysis was restricted to those who had never smoked; however, no association was apparent with antibody levels to *Actinobacillus actinomycetemcomitans*. In the most comparable study of a free-living population of never smokers, our finding of tooth loss being unrelated to myocardial infarction was replicated, although effect estimates were not stratified by gender.^12^ Some support for our observation of no effect is also found in analyses of a cohort of students followed for several decades from university entry. At the time of assessment of smoking in late adolescence/early adulthood, which would have been around the period of initiation of the habit, it is unlikely that smoking would have had a deleterious influence on oral disease, so breaking the confounding structure.^24^ In that study there was also no apparent tooth loss–coronary heart disease relationship.

### Study strengths and limitations

While our study has a series of strengths, not least its size, which allowed us to explore the link between tooth loss and coronary heart disease in a very large group of never smokers and the use of health professional-ascertained information on tooth loss, it is not without its limitations. First, we only had one indicator of oral health. The use of others – bleeding on probing and pocket depth – would have allowed us to test convergence of evidence. We dropped some study members because of missing data. The characteristics of excluded study members relative to those in the analytical sample revealed that the absolute difference in the characteristics between the groups was small but achieved statistical significance at conventional levels because of the large numbers of people. Tooth loss is a time-dependant variable, such that its prevalence increases with age; however, our analyses are based on a single baseline assessment. In addition, some study members will have lost teeth for reasons other than oral disease, including trauma, which, although likely to be relatively rare, was not captured during the oral examination here. It is also the case that the resolution of data for some potential covariates was modest. That is, it was not possible, for instance, to identify life-long never drinkers as it was to identify life-long never smokers. Lastly, other analyses of the present data have revealed known associations for blood glucose and cardiovascular disease,^25^ smoking and cancer,^26^ body mass index and mortality,^13^ among others. This therefore gives us some confidence in our present results for tooth loss.

In conclusion, on the basis of results from the present study, in men but not women, the modest tooth loss–coronary heart disease gradient appeared to be explained by confounding by cigarette smoking. Other sufficiently well powered studies are required to replicate these findings.

## Supplemental Material

Supplemental material for Oral health and later coronary heart disease: Cohort study of one million people Click here for additional data file. Supplemental material for Oral health and later coronary heart disease: Cohort study of one million people by G David Batty, Keum Ji Jung, Yejin Mok, Sun Ju Lee, Joung Hwan Back, Sunmi Lee and Sun Ha Jee in European Journal of Preventive Cardiology
