# Supplementary material for: Oral health and later coronary heart disease: Cohort study of one million people
Source: Eur J Prev Cardiol. 2018 Feb 20;25(6):598–605. doi: 10.1177/2047487318759112 (PMC5946673; doi:10.1177/2047487318759112)
Supplement: Supplemental material for Oral health and later coronary heart disease: Cohort study of one million people [file Supplemental_material.pdf]

Supplemental Figure 1. Age-adjusted hazard ratios (95% confidence intervals) for the relation of tooth loss with coronary heart disease in the Korean Cancer Prevention Study

A. Men (N=626,106)

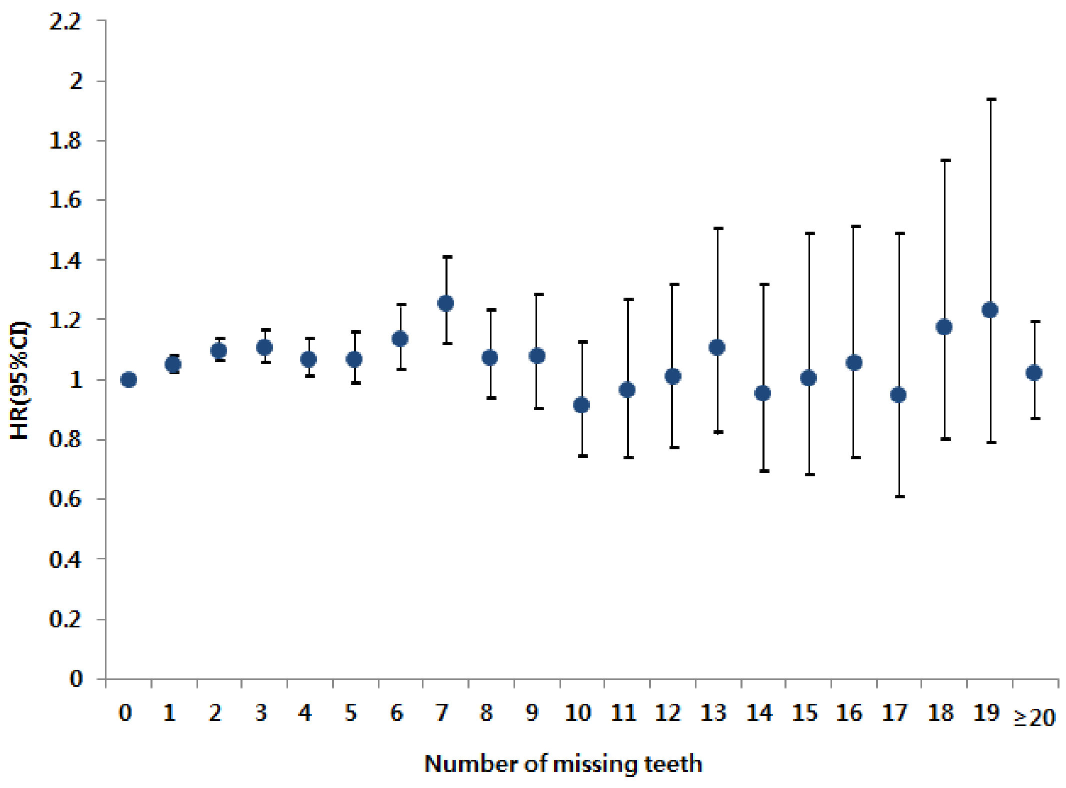

B. Women (N=349,579)

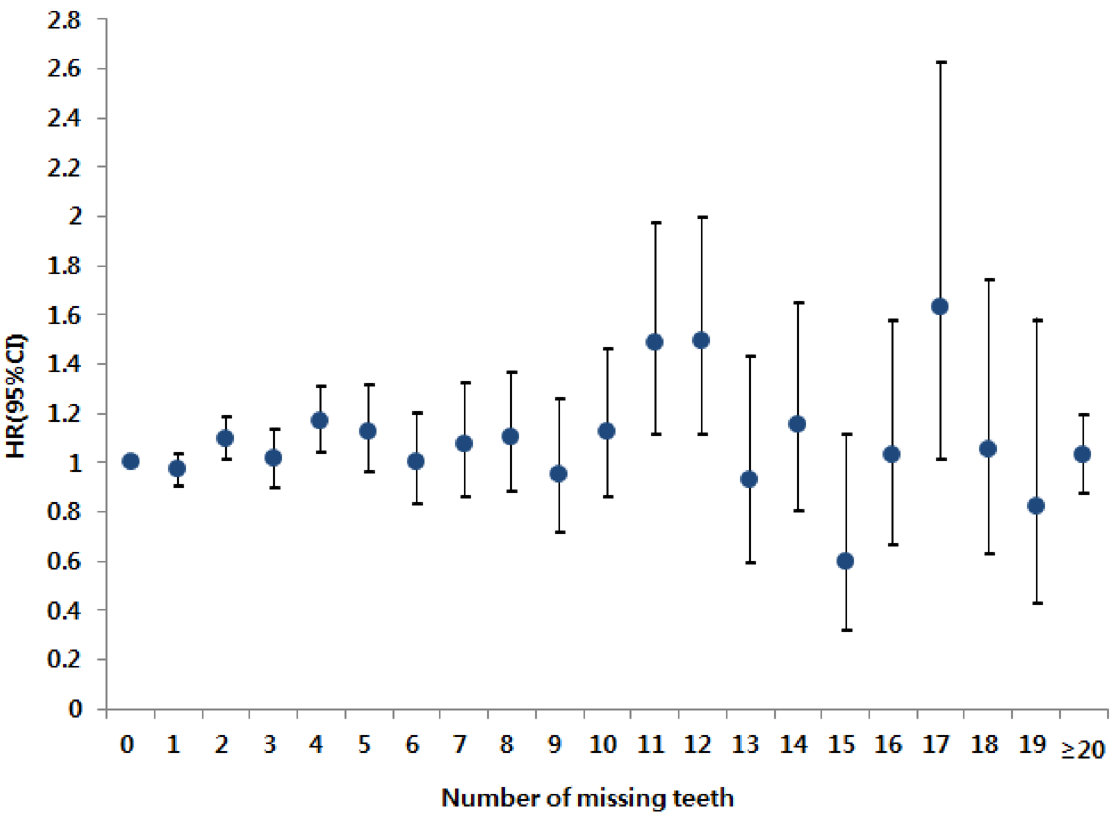

Supplemental Table 1. Baseline characteristics of study members included and excluded from the analytical sample: 1,329,525 men and women in the Korean Cancer Prevention Study

	**Includees (N=975,685)**	**Excludees (N=353,840)**
Age (year), mean (SD)	46.1 (11.0)	49.0 (13.2)
Tooth loss, mean (SD)	0.7 (2.2)	0.8 (2.6)
Men, percent (N)	64.2 (626,106)	62.4 (220,801)
SES (insurance contribution, Won x 10^3), mean (SD)	114.1 (73.5)	116.1 (79.1)
Height (cm), mean (SD)	163.9 (8.5)	162.6 (9.0)
Alcohol intake (g/day), mean (SD)	11.1 (26.7)	10.6 (27.8)
Current smoker, % (n)	38.6 (376,239)	39.5 (139,747)
No Exercise, % (n)	75.4 (735,214)	76.2 (256,413)
Systolic blood pressure (mmHg), mean (SD)	123.0 (16.9)	125.9 (19.4)
Fasting blood cholesterol (mg/dL), mean (SD)	192.5 (38.1)	192.7 (39.9)
Diabetic, % (n)	4.2 (40,679)	6.1 (21,682)
Body mass index (kg/m^2), mean (SD)	23.2 (2.8)	23.2 (3.0)
Family history of coronary heart disease, % (n)	16.5 (136,270)	16.7 (48,647)
Coronary heart disease rate (100,000 person-years)	343.8	387.2
Age-adjusted hazard ratio (95% confidence interval) coronary heart disease	1.0 (ref)	1.08 (1.06, 1.09)
